# A non-separation diagnostic framework for assessing canine attachment structure

**DOI:** 10.3389/fvets.2026.1802205

**Published:** 2026-04-17

**Authors:** Jongkyu Kim

**Affiliations:** Korean Native Dog Conservation and Research Center, Inje, Republic of Korea

**Keywords:** animal behavior, canine attachment, diagnostic framework, human-canine bond, non-separation diagnosis, separation anxiety, welfare-oriented assessment

## Abstract

Conventional diagnostic approaches to canine attachment and separation-related behaviors rely primarily on behavioral outcomes observed during induced separation from the caregiver. While widely adopted, separation-based assessments may conflate superficially similar behaviors that arise from fundamentally different caregiver–dog relational structures (e.g., secure regulation vs. avoidant disengagement vs. ambivalent hyper-proximity), thereby raising ethical, conceptual, and interpretive limitations. This study proposes a non-separation diagnostic framework for assessing canine attachment structure through everyday interactional observation, without inducing caregiver absence. Drawing on long-term naturalistic observation of dogs living in stable human environments and structured non-separation observation of representative cases, attachment-related organization was consistently observed as identifiable prior to any separation event. Diagnostic assessment focused on exploration–return organization (i.e., exploratory expansion followed by repeated voluntary returns to caregiver proximity as a regulatory anchor), distance regulation patterns, emotional stabilization following return, and caregiver emotional responsiveness. Across observed cases, attachment structures were consistently observable under non-separation conditions. Behaviors later expressed during separation were found to reflect amplification of pre-existing relational organization rather than responses generated by separation itself. Two procedural cases are presented to illustrate the diagnostic necessity of this framework. In the first, a presentation superficially resembling secure attachment was procedurally excluded due to absence of exploration and failure of emotional regulation. In the second, overt separation-related distress was observed, yet secure attachment organization was supported through intact non-separation structure. Together, these cases demonstrate that neither proximity nor separation behavior alone can serve as diagnostic criteria. The proposed framework repositions separation as an expressive context rather than a causal diagnostic trigger and establishes non-separation observation as the primary context for attachment classification. This approach offers a conceptually coherent and ethically grounded diagnostic alternative and provides a methodological foundation for subsequent applied and clinical studies of canine attachment.

## 1 Introduction

Attachment theory has provided a foundational framework for understanding relational structures between caregivers and dependent individuals across species (1, 3). In canine behavioral science, diagnostic assessment of canine attachment and separation-related behavior has historically relied on behavioral outcomes observed during physical separation from the caregiver. Vocalization, destructive behavior, and inappropriate elimination during caregiver absence have been treated as primary diagnostic indicators, forming the basis of most contemporary separation-related assessment protocols (4–7).

Empirical work has demonstrated that dogs display attachment-like behaviors toward human caregivers within modified Strange Situation Test (SST) procedures (2, 8, 9). Subsequent studies have further examined human–dog attachment dynamics across diverse contexts and methodological variations (10, 11). While separation-based approaches have contributed to clinical recognition of attachment-related difficulties, they embed a critical conceptual assumption: that separation itself functions as the primary causal trigger of anxiety-related behavior. This assumption has led to widespread interpretation of separation-related behaviors as direct expressions of anxiety, despite accumulating evidence that similar behavioral expressions may arise from distinct relational, developmental, and communicative structures (12).

For example, sustained proximity may reflect secure regulatory anchoring characterized by flexible exploratory expansion and rapid recovery following minor perturbations, whereas comparable proximity may also reflect ambivalent hyper-proximity associated with persistent vigilance, inhibited exploration, and heightened sensitivity to caregiver micro-movements. Likewise, low overt distress during separation may reflect secure coping in some dyads but avoidant disengagement in others, marked by reduced caregiver-directed signaling and limited reunion repair behaviors.

Several limitations emerge from this outcome-focused paradigm. First, separation-based assessment conflates heterogeneous attachment organizations that may appear phenotypically similar during caregiver absence but differ fundamentally in underlying relational structure (13, 14). Second, diagnostic inference is deferred until separation occurs, obscuring the possibility that attachment organization may already be identifiable under everyday interactional conditions. Third, induced separation raises ethical and methodological concerns, particularly when applied repeatedly or used as a routine diagnostic trigger rather than an investigative exception (6, 7). Ethically, induced separation may impose avoidable distress in clinical populations already presenting with fear, anxiety, or aggression. Methodologically, separation can function as a high-intensity arousal trigger that amplifies expressive stress behaviors (e.g., vocalization, locomotor agitation), potentially obscuring relational structure by elevating situational reactivity and increasing interpretive ambiguity in borderline cases (12, 15).

The present study addresses this conceptual gap by proposing an alternative diagnostic logic centered on non-separation procedural convergence. Rather than initiating caregiver absence as the primary diagnostic trigger, this framework evaluates relational structure through graded observational stages conducted within everyday indoor environments that approximate routine living contexts.

This Methods article proposes a structured clinical diagnostic architecture for assessing canine attachment under non-separation conditions. The present report emphasizes procedural transparency and conceptual logic. Formal validation, including inter-rater reliability quantification and systematic comparison with separation-based paradigms such as the SST (2, 16), is planned as subsequent empirical work.

## 2 Materials and methods

### 2.1 Study population and developmental corpus

This study adopts a qualitative, procedure-based diagnostic framework grounded in non-separation observational analysis. The primary objective is to articulate and illustrate a method for assessing canine attachment structure without inducing caregiver absence. Accordingly, the study does not include experimentally induced separation, behavioral intervention, or treatment evaluation.

Observations encompassed more than 180 dog–caregiver dyads evaluated between March 2023 and early 2026 within a structured canine behavioral intervention program operated by the author since 2017. These cases formed the developmental foundation from which the diagnostic architecture was iteratively refined. Over the past 4 years, more than 100 unedited video recordings have been systematically archived with participant consent, providing additional retrospective verification material.

The observed sample reflects a problem-behavior referral population rather than a community-based sample. As of December 2025, approximately 60% of cases were classified as secure attachment, 20% as ambivalent attachment, 17% as avoidant attachment, and 3% as disorganized attachment. These proportions are reported descriptively to characterize the referral population observed in this clinical context and do not represent statistical inference, prevalence estimates, or population-level generalization.

The present manuscript does not aim to provide statistical generalization. Instead, the observational corpus functioned as a structural reference base for refining procedural exclusion and convergence logic. The two cases presented were intentionally selected as procedural boundary exemplars to illustrate diagnostic logic rather than prevalence.

### 2.2 Observation procedure

Observations were conducted primarily through live assessment during structured behavioral consultation sessions. Each dyad participated in a minimum of two full-length sessions, each lasting at least 60 min. In cases involving aggression or complex behavioral presentation, extended programs consisting of eight or more sessions were conducted to ensure procedural stability.

Observational environments consisted of indoor consultation or training spaces approximating routine daily living contexts. These were structured but not laboratory-standardized experimental rooms, thereby maintaining basic environmental consistency (including defined indoor spatial boundaries and controlled human presence) while minimizing novelty stress. No experimental separation induction or stress-eliciting manipulation was introduced.

As a procedural condition, dogs entered wearing a harness and leash; the leash was removed before observation began (the harness remained on). During assessment sessions, dogs were off-leash and free to move within the observation environment.

Caregivers were not instructed to follow scripted behavioral procedures during the observation. Interaction between the caregiver and the dog was allowed to unfold naturally. Only minimal behavioral instructions were provided: caregivers were asked to sit on the floor rather than on a chair to maintain physical accessibility to the dog, and they were asked not to intervene in or restrain the dog's behavior during the observation. These conditions were introduced to ensure naturalistic interaction while maintaining a consistent observational context. The diagnostic steps (Steps 1–7) therefore represent analytical stages of observational interpretation, not behavioral procedures imposed on the participants. In particular, “rest” (Step 3) was coded as an observed outcome (i.e., the dog settling into a relaxed posture near the caregiver) rather than a caregiver-instructed action.

Proximity distances (approximately 30 cm and 1 m) were estimated visually using spatial reference markers within the consultation environment, such as floor tiles and fixed furniture alignment. These thresholds functioned as functional relational indicators rather than precise geometric measurements.

Caregiver–dog proximity was interpreted using three functional relational distance categories. Contact proximity (within approximately 30 cm) corresponds to the range within which the caregiver can easily reach and physically hold the dog (immediate reach distance). Close regulatory proximity (approximately 30 cm−1 m) represents a distance at which the caregiver can reach the dog but the dog remains outside immediate holding distance, allowing the dog to maintain minimal regulatory autonomy. Distances beyond approximately 1 m represent a range in which immediate physical contact is not possible; sustained maintenance of this distance was often observed in avoidant-like presentations in this clinical context.

These categories represent observational operational definitions derived from repeated clinical observation rather than precise geometric measurements or standardized metrics from existing literature.

### 2.3 Caregiver emotional rhythm assessment

Prior to interpreting canine behavior, caregiver emotional rhythm was assessed as a contextual diagnostic variable. Emotional rhythm was operationalized across three domains:

- *Consistency*, defined as stable attentional monitoring and predictable response patterns.
- *Sensitivity*, defined as the ability to detect and appropriately interpret the dog's gaze, orientation, and early emotional signals.
- *Immediacy*, defined as timely response when the dog exhibited startle, fear, freezing, or dysregulation.

Caregiver Emotional Rhythm indicators were evaluated through structured clinical interview and protocol-based observation of caregiver–dog micro-interaction patterns during entry, positioning, voice modulation, and response timing. Presence of coercive control, punitive interaction, or fear-inducing cues was additionally noted, as these factors elevate risk for disorganized attachment. Caregiver variables were treated as contextual conditions necessary for interpreting relational organization (3).

### 2.4 Diagnostic architecture and observation protocol

The proposed diagnostic framework consists of sequential procedural stages designed to evaluate attachment structure without initiating caregiver separation. A schematic representation of the procedural diagnostic logic is provided in Supplementary Figure S1.

#### 2.4.1 Analytical strategy and operational indicators

Classification was derived from protocol-guided clinical observation of (i) entry sequence and caregiver-following organization, (ii) proximity regulation to a seated caregiver (approximately within 30 cm−1 m vs. sustained distance beyond 1 m), (iii) exploration–return organization (gradual expansion with repeated voluntary return vs. expansion without repair or return), (iv) response to a highly palatable “litmus-level” treat placed neutrally (consumption, approach without consumption, or refusal), (v) response to passive unfamiliar human presence (regulated approach-and-check vs. sustained vigilance or disengagement), and (vi) response to caregiver micro-movements and vocal modulation (rapid regulatory recovery vs. persistent hyper-monitoring or non-repair disengagement). Classification required procedural convergence across repeated contexts and sessions rather than reliance on any single behavioral stimulus.

#### 2.4.2 Operational clarification

Terms such as “rapid recovery,” “sustained vigilance,” and “regulatory stabilization” refer to observable behavioral transitions rather than internal states. “Rapid recovery” was defined as visible reduction in motor tension (e.g., cessation of pacing, reduction of scanning head movements, relaxed posture, re-engagement in exploratory behavior) within the same observational phase following caregiver vocal or positional cue. “Sustained vigilance” referred to persistent environmental scanning, muscular stiffness, fixed gaze, or delayed exploratory expansion across multiple minutes of observation. “Regulatory stabilization” was operationalized as expansion of exploration accompanied by repeated voluntary returns to caregiver proximity without escalation of motor tension or distress signals.

#### 2.4.3 Attachment classification criteria

Attachment classification was determined through the convergence of behavioral indicators observed during the non-separation assessment protocol.

Secure attachment organization was identified when the following conditions were consistently observed:

1. The dog voluntarily maintained contact proximity or close proximity within approximately 30 cm to 1 m of the caregiver during baseline observation.
2. The dog demonstrated repeated exploration–return cycles, voluntarily moving away from and returning to caregiver proximity.
3. Exploratory behavior progressively expanded until salient areas of the environment were inspected.
4. After exploration, the dog maintained low-level attentional orientation toward the caregiver while remaining behaviorally relaxed.
5. Following exploration, the dog returned to and remained in contact proximity or within approximately 30 cm to 1 m of the caregiver in a calm and regulated resting state.

If one or more of these criteria were incomplete or ambiguous, a secondary diagnostic assessment was conducted using the dog's response to an unfamiliar person and food offered by that unfamiliar person. Secure attachment organization was supported when the dog demonstrated initial vigilance followed by exploratory approach and gradual relaxation toward the unfamiliar person, including acceptance of food after exploratory investigation.

Persistent avoidance or unconditional approach without exploratory investigation was interpreted as evidence supporting insecure attachment organization.

Other attachment patterns (avoidant, ambivalent, and disorganized) were identified through the differential behavioral patterns described in Step 7 (Differential Filter) of the diagnostic protocol (see below).

The procedure-based framework proposed here addresses common diagnostic errors by treating attachment classification not as a judgment of behavioral appearance, but as the outcome of sequential procedural constraints. Secure attachment organization is not defined by proximity *per se*, but by the successful completion of a specific regulatory sequence: adaptive proximity regulation, emergence of exploration, progressive expansion of exploratory range, return to the caregiver, and observable emotional stabilization following return. Cases that fail to complete this sequence are procedurally excluded from secure classification. These procedural elements were implemented as protocol-guided clinical observation steps and are presented here to clarify reproducibility of the framework. These indicators are summarized in Table 1.

**Table 1:** Operational indicators for non-separation attachment assessment.

Observation sequence	Behavioral indicator	Interpretation
Entry response	Dog follows caregiver into the environment	Caregiver-oriented entry behavior
Baseline proximity	Dog maintains contact proximity or 30 cm−1 m proximity	Regulatory proximity
Exploration initiation	Dog moves away from caregiver to explore	Exploratory activation
Exploration–return pattern	Dog voluntarily returns to caregiver proximity	Secure base behavior
Post-return regulation	Visible reduction of tension after return	Emotional stabilization
Repeated cycles	Exploration–return pattern repeats	Organized regulation pattern
Differential assessment	Response to unfamiliar person and food	Attachment classification according to Step 7

The following steps describe the sequential observational stages through which these indicators are evaluated.

**Steps 1–3: Baseline Relational Stability**

Initial stages assess spontaneous proximity maintenance, gaze orientation, and caregiver-directed behavioral regulation under neutral conditions.

**Steps 4–5: Mild Relational Perturbation**

Graded contextual variations (e.g., caregiver positional shift, neutral environmental distraction) are introduced to observe regulatory adaptation without stress induction.

**Step 6: Procedural Convergence Assessment**

Attachment classification logic emerges when relational responses demonstrate structural consistency across graded contexts.

**Step 7: Differential Diagnostic Filter**

Step 7 was operationalized through low-intensity contextual probes designed to distinguish superficially calm or proximity-maintaining dogs from structurally secure dyads. Within Step 7, structured observation of the dog's approach pattern toward a passive unfamiliar person was conducted under caregiver presence. Incidental brief caregiver absences (e.g., restroom use) were not treated as protocol steps but as naturally occurring events. Classification did not rely solely on whether the dog approached, but on the sequence, regulatory quality, and food-response pattern associated with that approach.

Dogs exhibiting secure attachment organization typically demonstrated an initial period of vigilance and environmental scanning, followed by cautious proximity, olfactory investigation, and progressive behavioral relaxation. Once regulatory safety appeared established, these dogs accepted a highly palatable food item and frequently displayed subsequent affiliative or food-seeking behavior toward the unfamiliar person. This sequence reflected exploratory expansion supported by caregiver-based regulatory security.

Avoidant presentations manifested in two distinct patterns. Some dogs approached rapidly with exaggerated affiliative or appeasement-like gestures but demonstrated minimal food interest unless under strict feeding control; such behavior was interpreted as non-aggression signaling rather than relaxed exploratory engagement. Other avoidant cases demonstrated minimal approach, limited exploratory behavior, and reduced interaction with both the unfamiliar person and the environment.

Ambivalent presentations were characterized by sustained vigilance toward the unfamiliar person, rare spontaneous approach, and approach occurring primarily after repeated exposure across sessions. Unlike secure organization, exploratory expansion did not progress fluidly following initial contact.

Within Step 7, disorganized outcomes were considered when the dog displayed inconsistent regulatory responses during the unfamiliar-person probe, including simultaneous proximity-seeking and fear responses toward the caregiver; this pattern is consistent with disorganized constraint pathways identified at earlier stages (Steps 1 and 5) and shown in Supplementary Figure S1.

The treat-response indicator was not interpreted in isolation. Food refusal was considered meaningful only when occurring alongside convergent behavioral patterns (e.g., motor tension, inhibited exploration, persistent vigilance). Individual variation in appetite, food preference, and novelty response was therefore contextualized within the broader procedural sequence rather than treated as a standalone diagnostic marker. Accordingly, Step 7 functioned exclusively as a differential safeguard to prevent premature secure classification in cases where baseline calmness or proximity alone might obscure avoidant disengagement or ambivalent hyper-vigilance, and not as a stress-induction trigger.

### 2.5 Evaluators and reproducibility

Diagnostic evaluations were conducted by the author, a nationally certified Companion Animal Behavior Instructor (Certificate No. B25200225, Ministry of Agriculture, Food and Rural Affairs, Republic of Korea) and licensed Livestock Artificial Insemination Technician (License No. 2577, Gyeonggi Province), with over three decades of longitudinal observational experience in Korean native dogs and 9 years of clinical experience in canine artificial insemination and semen cryopreservation. This background includes extensive non-invasive physiological handling of dogs under sensitive conditions, supporting stable behavioral assessment and minimizing stress-induced behavioral artifacts.

The diagnostic architecture was systematically developed over approximately 8 years of repeated clinical application and observational work, with progressive clarification of exclusion boundaries and reduction of interpretive ambiguity.

Caregiver-reported separation behaviors were obtained during initial consultations through structured clinical interviews combined with clinical observation grounded in the developed non-separation diagnostic protocol. No standardized questionnaire instruments were used.

A standardized diagnostic flowchart and structured observation sheet were used during parallel documentation by a trained evaluator who completed the author-developed behavioral specialist training program. Independent concurrent documentation procedures were implemented to support procedural reproducibility. In a subset of 42 cases independently documented by the author and the trained rater, agreement on categorical outcomes reached 86% (Cohen's κ = 0.78), indicating substantial inter-rater reliability. Expanded reliability testing (e.g., across additional evaluators and independent sites) is planned as a subsequent validation phase. This article presents a methodological framework derived from structured clinical observation rather than a formal statistical validation study.

### 2.6 Ethical considerations

This study was conducted in accordance with the local legislation and institutional requirements. Ethical review and approval were not required because the study involved only non-invasive observation of client-owned dogs during routine behavioral consultations, without any experimental manipulation or modification of the dogs' environment. Written informed consent was obtained from the owners for the participation of their animals in this study and for the use of their data for research purposes.

## 3 Results

Across more than 180 observed dog–caregiver dyads, attachment classification decisions were derived through procedural exclusion and convergence rather than separation-induced stress behavior. A procedural summary of representative diagnostic trajectories is presented in Table 2.

**Table 2:** Procedural case mapping summary.

Case	Observed pattern	Procedural outcome
Case 1	High proximity, absent exploration, no regulatory stabilization	Procedural exclusion from secure classification
Case 2	Adaptive proximity, progressive exploration, regulatory return, rapid post-separation recovery	Procedural convergence consistent with secure attachment organization

In follow-up observations exceeding 6 months (n = 62; all followed for ≥ 1 year), secure attachment classifications remained stable across all previously secure cases (36/36). Structural transitions were observed in 14 of 26 ambivalent or avoidant cases over time; however, detailed evaluation of post-diagnostic clinical interventions exceeds the scope of the present methodological report.

The two cases presented below represent procedural boundary exemplars selected from the broader observational corpus to illustrate diagnostic exclusion and convergence logic rather than representing the entirety of assessed cases.

### 3.1 Procedural exclusion case: pseudo-secure presentation under non-separation conditions

Case 1 (Dog BO; anonymized case code) was a 4-year-old spayed female Welsh Corgi rescued from an animal shelter with suspected prior maltreatment. She lived with a female caregiver, following a period of household instability and familial conflict regarding dog care.

Under non-separation conditions, the dog remained within 30 cm of the caregiver at all times and displayed sustained visual attention toward the caregiver. Superficially, this presentation resembled secure attachment. However, no exploratory behavior emerged throughout observation. The dog did not initiate environmental exploration, nor did exploration appear with time. Presentation of a litmus-level treat (a highly palatable food item that most dogs in this clinical context are unlikely to refuse unless conflict, inhibition, or dysregulation is present) elicited no approach or investigation. Unfamiliar individuals were ignored. Caregiver movement, changes in vocal tone, or presentation of salient objects elicited anxiety and panic responses that did not resolve through proximity.

Despite continuous caregiver presence, emotional rhythm failed to stabilize. Proximity did not function as a regulatory mechanism. Based on procedural criteria—absence of exploration, failure of emotional regulation, and rigid proximity—secure attachment organization was explicitly excluded. The attachment structure was classified as ambivalent with elevated risk of disorganized progression, despite superficial markers commonly misinterpreted as secure attachment.

### 3.2 Procedural convergence case: apparent separation distress with secure attachment organization

Case 2 (Dog TA; anonymized case code) was a 3-year-old neutered male Jindo-mix living with two female caregivers. Only one caregiver participated in the consultation sessions. At least one caregiver was consistently present in daily life. No history of neglect or abuse was reported.

Under non-separation conditions, the dog followed the caregiver upon entry, settled within approximately 1 m, and initiated exploration after brief acclimation. Exploration–return cycles were repeated, with gradual expansion of exploratory radius. Following completion of exploration, the dog rested calmly while maintaining attentional orientation toward the caregiver. The dog readily consumed a litmus-level treat and approached unfamiliar individuals with exploratory interest, accepting food after brief assessment. Sudden environmental stimuli elicited brief startle responses followed by rapid recovery.

During consultation, the caregiver briefly left the room for restroom use. The dog attempted to follow and emitted vocalizations during the approximately 2-min absence. Upon caregiver return, greeting and soothing contact resulted in emotional stabilization within approximately 1 min.

Despite overt separation-related vocalization and following behavior, procedural evaluation converged on secure attachment organization due to intact exploration–return structure, effective emotional recovery, and stable caregiver responsiveness under non-separation conditions.

Together, the procedural exclusion and procedural convergence cases demonstrate that attachment diagnosis cannot be derived from proximity or separation behavior alone. In this framework, procedural evaluation provides a structured basis for attachment classification by requiring convergence across multiple low-intensity contexts and repeated sessions, thereby reducing over-reliance on any single expressive behavior. Classification was determined when the observed indicator pattern remained internally consistent across Steps 1–6 and, when required, passed Step 7 as a differential filter against avoidant or ambivalent structures.

## 4 Discussion

The proposed non-separation diagnostic architecture challenges the assumption that separation response constitutes the primary structural indicator of attachment classification. Rather than relying on stress expression during caregiver absence, this framework evaluates attachment structure through graded relational regulation within ecologically valid contexts. This framework does not negate the clinical relevance of separation responses but aims to distinguish expressive stress reactions from structural relational organization.

### 4.1 Why a procedural diagnostic framework is necessary

The present study was motivated by a persistent diagnostic problem in canine attachment assessment: surface behaviors that appear affiliative or proximity-oriented under non-separation conditions are frequently misclassified as indicators of secure attachment. The most common error involves interpreting sustained closeness to the caregiver as evidence of attachment security without evaluating whether such proximity performs a regulatory function. Proximity may function as a regulatory context supporting exploratory flexibility and affect stabilization, consistent with secure base theory and canine attachment research (1, 2, 10).

The following discussion focuses on conceptual implications of convergence-based classification rather than repeating procedural details described in Methods.

### 4.2 The function of procedural exclusion: case 1 as a minimal illustrative case

Case 1, intentionally positioned as the first illustrative case in this study, demonstrates the diagnostic power of procedural exclusion. Under non-separation conditions, the dog in Case 1 remained within 30 cm of the caregiver, displayed sustained visual monitoring, and never disengaged spatially. However, when evaluated through the non-separation procedure, the case failed at multiple critical steps. Exploratory behavior was absent, and sustained proximity did not result in emotional stabilization. Procedurally, this case was therefore excluded from secure attachment classification despite high proximity and apparent attentiveness.

### 4.3 Procedural convergence and secure organization: case 2 as a contrasting illustration

Case 2 provides a contrasting illustration of procedural convergence consistent with secure attachment organization. In this case, caregiver emotional response rhythm satisfied all prerequisite conditions. Under non-separation observation, the dog demonstrated adaptive proximity regulation, progressive exploration, and clear emotional stabilization following return. Notably, when an unintended separation occurred, the dog vocalized and attempted to follow. However, upon reunion, emotional regulation was rapidly restored. This sequence suggests that separation behavior may function as an expressive context rather than a primary causal diagnostic trigger. This interpretation is based on repeated observations that similar separation-linked behaviors (e.g., vocalization, locomotion, protest) occurred in dyads with markedly different non-separation relational organization, suggesting that separation responses may reflect situational arousal rather than uniquely determining attachment structure.

### 4.4 Distinguishing this framework from separation anxiety reclassification

It is critical to delineate the scope of the present framework from separation anxiety reclassification models. This study does not diagnose separation anxiety, redefine separation-related syndromes, or evaluate treatment outcomes. Instead, it establishes a prior diagnostic layer: the identification of attachment organization under non-separation conditions. This distinction is necessary because non-separation structural classification and separation-triggered risk assessment address different clinical questions: the former targets relational organization under everyday interaction, whereas the latter may index stress reactivity under induced absence.

### 4.5 Ethical–methodological implications

Ethical and methodological issues are closely coupled, as procedures that elevate distress may simultaneously alter behavioral expression and confound interpretation. This framework avoids reliance on stress induction as a diagnostic tool and eliminates the need to provoke distress for diagnostic clarity.

### 4.6 Limits of classification and the principle of diagnostic restraint

A defining feature of this framework is its conservative stance toward classification. When procedural convergence is insufficient, attachment classification is deferred. This restraint is intentional to avoid over-interpreting ambiguous data. In practice, deferred classification refers to postponing categorical assignment when procedural indicators remain internally inconsistent across sessions. In such cases, the case is recorded as indeterminate and scheduled for additional observation rather than forcing premature classification from a single presentation.

### 4.7 Limitations and future directions

This study has several limitations. First, while preliminary inter-rater reliability data are reported (Section 2.5), the protocol remains developmental and has not yet undergone formal quantitative validation or systematic comparison with established separation-based paradigms such as the Strange Situation Test. Second, classification decisions were derived from qualitative structural analysis rather than predefined numerical thresholds. Third, reliability data were derived from a single clinical site; replication across independent clinical cohorts remains necessary. Fourth, while preliminary longitudinal follow-up observations exceeding 1 year demonstrate stability, formal predictive validity analysis, including statistical modeling of structural progression, exceeds the scope of the present methodological report and will be addressed in future publications. Future research will include systematic validation studies comparing non-separation diagnostic outcomes with separation-based paradigms (16), formal inter-rater reliability quantification, predictive stability modeling, and direct comparative analyses.

## 5 Conclusion

This study proposes a non-separation, procedure-based diagnostic framework for assessing canine attachment structure and suggests that attachment organization may be identifiable prior to any separation event. By defining attachment types as outcomes of sequential procedural constraints, the framework addresses a central diagnostic ambiguity in canine attachment assessment. The framework is intentionally diagnostic rather than therapeutic, establishing the structural conditions necessary for valid interpretation of attachment-related behavior. Direct quantitative correspondence with separation-based classifications such as the Strange Situation Test has not yet been formally examined in this report. Future empirical research will focus on formal quantitative validation, including expanded inter-rater reliability assessment, predictive modeling, and direct comparative analyses.

## Data Availability

The raw observational notes and consultation records contain potentially identifiable information and are therefore not publicly available. De-identified excerpts supporting the conclusions of this article will be made available by the authors upon reasonable request.
