# Supplementary material for: A non-separation diagnostic framework for assessing canine attachment structure
Source: Front Vet Sci. 2026 Apr 17;13:1802205. doi: 10.3389/fvets.2026.1802205 (PMC13132736; doi:10.3389/fvets.2026.1802205)
Supplement: Supplementary file 1 [file Image_1.pdf]

Supplementary Figure S1. Non-Separation Diagnostic Architecture

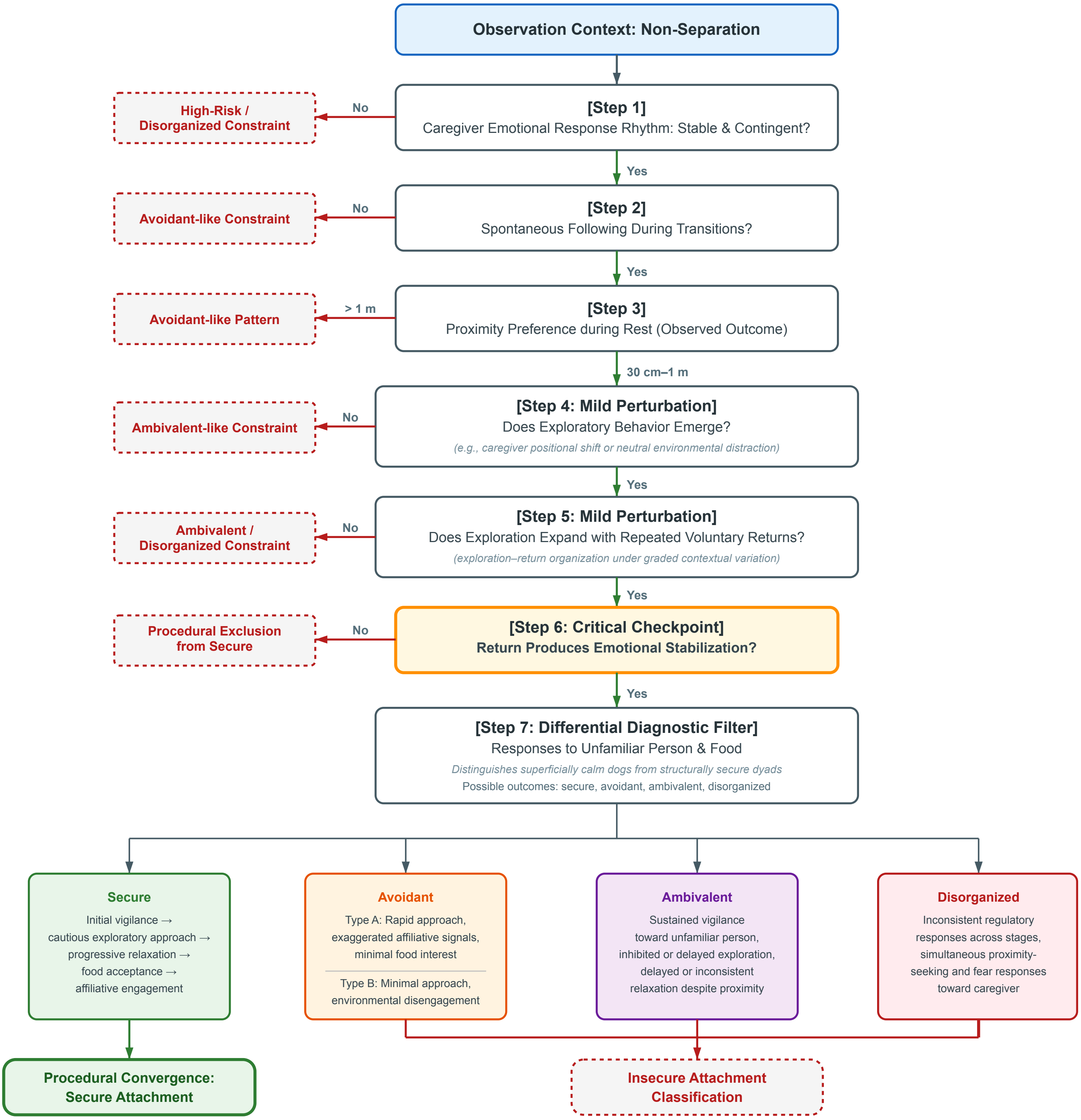

Steps 1–3 assess baseline relational stability. Steps 4–5 introduce mild perturbation to observe regulatory adaptation.
Step 6 evaluates whether return produces emotional stabilization. Step 7 functions as a differential diagnostic filter.
All diagnostic steps represent analytical stages of observational interpretation, not behavioral procedures imposed on participants.

Secure pathway	Exclusion pathway	Critical checkpoint
Progression	Exclusion / Risk	Differential outcome

Supplementary Figure S1. Schematic representation of the non-separation diagnostic architecture. Steps 1–6 evaluate relational stability and regulatory organization across graded observational contexts. Step 7 functions as a differential diagnostic filter designed to distinguish superficially calm proximity-maintaining dogs from structurally secure attachment organization. Possible interpretive pathways include: (a) secure attachment (regulated exploratory approach and progressive relaxation toward an unfamiliar person); (b) avoidant patterns (rapid affiliative signaling or disengaged distance maintenance without exploratory investigation); (c) ambivalent patterns (sustained vigilance and inhibited exploration with delayed or inconsistent relaxation despite proximity); and (d) disorganized patterns (inconsistent regulatory responses and simultaneous proximity-seeking and fear responses toward the caregiver). Incidental caregiver absences were considered naturally occurring events rather than protocol steps. All diagnostic steps represent analytical stages of observational interpretation, not behavioral procedures imposed on participants.
